# Mussel-Inspired Co-Deposition of Polydopamine/Silica Nanoparticles onto Carbon Fiber for Improved Interfacial Strength and Hydrothermal Aging Resistance of Composites

**DOI:** 10.3390/polym12030712

**Published:** 2020-03-23

**Authors:** Xuejun Cui, Lichun Ma, Guangshun Wu

**Affiliations:** 1 School of Chemistry and Materials Science, Ludong University, Yantai 264025, China; cuixj1964@163.com; 2 Institute of Polymer Materials, Qingdao University, Qingdao 266071, China; mlc@qdu.edu.cn

**Keywords:** polymeric composites, carbon fiber, surface modification, interface

## Abstract

A novel and effective strategy was first proposed for the codeposition of a mussel-inspired nanohybrid coating with excellent wettability onto the surface of carbon fibers (CFs) by simultaneous polymerization of bioinspired dopamine (DA) and hydrolysis of commercial tetraethoxysilane (TEOS) in an eco-friendly one-pot process. Mussel-inspired nanohybrids could be adhered onto the surface of CFs firmly. The novel modification could afford sufficient polar groups and significantly improve fiber surface roughness and energy without decreasing fiber intrinsic strength, which were advantageous to promote interfacial compatibility and wettability between CFs and matrix resin. As a result, the interfacial shear strength of composites increased to 48.21 ± 1.45 MPa compared to that of untreated composites 29.47 ± 0.88 MPa. Meanwhile, the nanohybrid coating increased significantly composites’ hydrothermal aging resistance. The efficient strategy shows a promising and green platform of surface functionalization of CFs for preparing advanced polymer composites arising from broadly mechanical-demanding and energy-saving usages.

## 1. Introduction

Carbon fibers (CFs) have become the ideal reinforcements for advanced polymer composites, which are widely used as structural materials in engineering applications of the automotive and aerospace industries, owing to their high strength-to-weight ratio, excellent heat or chemical resistance [1,2,3,4]. It is widely accepted that fiber-matrix interface plays a key role in connecting CFs with the hot resin and ensuring efficient stress transfer, which is critical factor for overall properties of polymer composites [5,6,7]. Unfortunately, the fiber smooth and inert surface results in non-ideal interfacial combination and wettability effect with matrix resin [8,9]. Hence, continuous endeavor on fiber surface grafting, like oxidation treatments [10], plasma etching [11], chemical grafting [12,13], polymer sizing [14,15] and high energy irradiation [16,17], has been devoted to improve the surface/interfacial structure and chemistry for preparing advanced polymer composites. However, the above techniques may have certain limitations, like multistep manipulation, high toxicity, high energy consumption, the usage of strong acids with decreasing fiber tensile strength, and catalyst processing. Hence, a facile, effective and green strategy which can realize the similar fiber surface activation and interfacial reinforcing effect without damaging the fiber intrinsic tensile strength is highly desirable.

**Figure 1:**
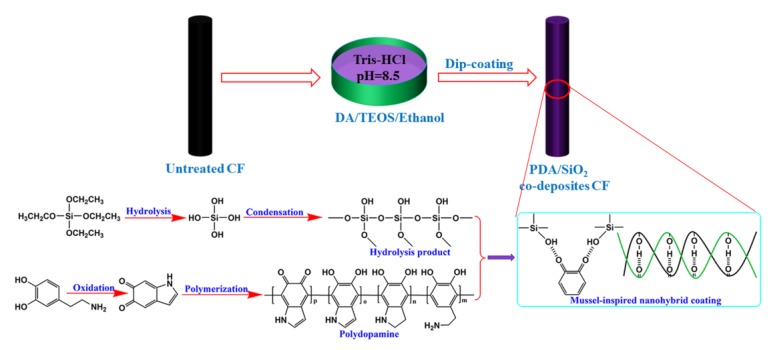
The schematic illustration for the immobilization of PDA/SiO_2_ nanohybrid coatings onto the fiber surface.

In particular, a mussel-inspired coating technique about dopamine (DA) was widely used to graft almost all types of substrates with the strong binding strength by self-polymerizing into polydopamine (PDA) coating owing to its stability, simplicity, and versatility [18,19,20]. The study on the modified surface of CFs with obvious changing surface/interface properties has been reported [21]. However, the introduced active groups by pure PDA coating were insufficient and unable to provide enough wettability and reaction between CFs and matrix resin because of the inherent and limited wettability of PDA with very few residual reaction groups after the polymerization process. Modified carbon fiber by silica nanoparticles can be considered to be an effective method for producing highly reinforced composites owing to its unique spherical molecular structure and the distinct price advantage [22,23]. Interfacial properties of advanced polymer composites could be obviously enhanced via self-polymerized polydopamine followed via the sol-gel growing of silica nanoparticles onto carbon Fiber [24]. However, the two-step methods for subsequent modifications onto PDA-based fibers via Michael addition and Schiff base reactions are time-consuming, complicated, low efficiency and high energy consumption [25]. Noteworthily, silica nanoparticles with abundant hydroxyl groups are easily obtained via the gradual hydrolysis and condensation of TEOS in an alkaline condition. The hydrophilic silica nanoparticles and PDA are deposited onto the fiber surface via hydrogen bonding and physical entanglement simultaneously in an alkaline aqueous solution to form mussel-inspired nanohybrid coatings with high stability and excellent wettability (Figure 1). Fiber microstructures, wettability, and composites interfacial strength as well as hydrothermal aging resistance were also studied systematically.

## 2. Materials and Methods

### 2.1. Materials

Carbon fibers (CFs, T300-3K) with the average diameter of round 7 μm were obtained from Toray Industries, Inc (Tokyo, Japan). Dopamine hydrochloride and Methylphenylsilicone resin (MPSR) with the viscosity of 25 cSt and the molecular weight of 2400 were provided by Sigma-Aldrich (Shanghai, China) and ShangHai Chemicals Co (Shanghai, China), respectively. Tris(hydroxyl-methyl)aminomethane (Tris) were purchased by Aladdin (Shanghai, China). All other chemical agents were used as received.

### 2.2. Fiber Surface Modification

PDA/SiO_2_ codeposited CFs (denoted as CF-PDA/SiO_2_) has been prepared by using the following typical process. First of all, ethanol (25 mL) containing the amounts of TEOS (0.30 g) were added to dopamine mixed aqueous solution (Tris-HCl, 100 mL, 2g/L, pH 8.5). Subsequently, the untreated CFs were quickly added into the above solution, and added to react by stirring for 9 h at room temperature. After that, the obtained fibers were washed many times by deionized water and ethanol separately. Figure 1 shows an illustration of the copolymerization of hybrid coatings onto the fiber surface.

### 2.3. Preparation of CF/MPSR Composites

Untreated and modified fibers reinforced MPSR composites were prepared by using unidirectional fiber prepreg based on the compression molding method, which is described in detail in the previous paper [13].

### 2.4. Characterization Techniques

Surface elements and morphologies of different CFs were tested by X-ray photoelectron spectroscopy (XPS, ESCALAB 220i-XL, VG, UK) and scanning electron microscopy (SEM, Quanta 200FEG, Hitachi Instrument, Inc., Tokyo, Japan), respectively. The dynamic contact angle meter (DCAT21, Data Physics Instruments, Stuttgart, Germany) was carried out to study dynamic contact angles and corresponding surface energies for examining fiber surface wettability. A single filaments tensile strength (TS) testing was carried out on an electronic universal testing machine (5569, Instron, Boston, MA, USA) according to ASTM D3379 with the cross-head speed of 10 mm/min and the gauge length of 20 mm [26]. A single fiber was separated from the carbon fiber bundles and attached to paper tabs at a distance of 20mm using an instant adhesive, and the paper strips were then cut off before the testing. Weilbull statistical method was carried out to analyze the testing results for 100 samples measurements for each fiber type. The interlaminar shear strength (ILSS) values of CF/MPSR composites were evaluated using a universal testing machine (WD-1, Changchun, China) in a three-point short-beam bending testing mode. Hydrothermal aging resistance of composites was characterized by tracing the changes in ILSS values with and without hydrothermal aging treatment in the boiling water for 48 h. The recorded value of ILSS and the hydrothermal aging resistance for each fiber type was averaged from 30 successful measurements data.

## 3. Results

### 3.1. Fiber Surface Composition and Microstructures

The chemical compositions of untreated and modified CFs were examined by XPS, as shown in Table 1. Untreated CF surface is mainly composed of carbon (C), oxygen (O) and a small amount of nitrogen (N). For CF-PDA, the intensities of N and O elements show the increasing trend after PDA coating compared with those of Untreated CF. After being grafted by PDA/SiO_2_, the increasing contents of O element (24.12%) and the new peak of Si element (6.83%) confirmed the successful deposition of PDA/SiO_2_ coating onto the fiber surface. In addition, the surface atomic O/C ratios of CF-PDA/SiO_2_ enhance to 0.10. The results indicate that codeposition of a mussel-inspired nanohybrid coating arising from the co-deposition of PDA and the hydrolysis product of TEOS simultaneously was realized.

**Figure 2:**
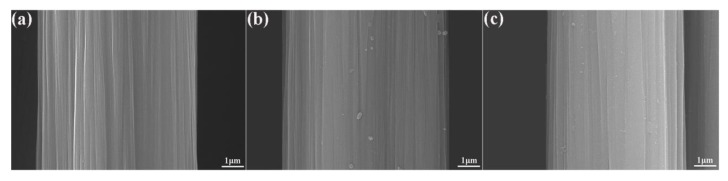
SEM images of different CFs surface: (**a**) untreated CF, (**b**) CF-PDA, and (**c**) CF-PDA/SiO_2_.

Surface topographies of different fibers are shown in Figure 2. It is widely accepted that the surface of Untreated CF (Figure 2a) is typically smooth and neat. After PDA deposition (Figure 2b), based on the nano-size thickness of PDA layer modified onto the surface of CFs, there are no significant differences of surface topographies between Untreated CF and CF-PDA according to SEM observation. However, the surface of resultant CF-PDA emerge some scattered nanoparticles due to the self-polymerized PDA. As for CF-PDA/SiO_2_, a very uniform layer of nanoparticles formed onto the fiber surface is obviously observed, and much more surface-bound nanoparticles are visible because the obtained SiO_2_ nanoparticles can immobilize into the PDA-based nanohybrid coating with the help of the favorable adhesion. The introduced PDA/SiO_2_ layer with high surface roughness and excellent interfacial wettability helps to improve the potential curing reactive area and the capacity of mechanical interlocking at the fiber-matrix interface.

### 3.2. Fiber Surface Energy and Wettability

The wettability of untreated and modified CFs was evaluated by the advancing contact angle and the surface energy, as shown in Table 2. The surface energy of the untreated fibers is 35.86 mN·m^−1^, with the dispersion component of 29.20 mN·m^−1^ and the polar component of 6.66 mN·m^−1^. For CF-PDA, the obvious decrease in contact angle and the great increase in surface energy may be due to the introduced many polar groups compared to those of Untreated CF. However, the contact angles decrease from 51.17° ± 1.54° to 41.89° ± 1.26° for water as well as from 49.89° ± 1.50° to 40.26° ± 1.21° for diiodomethane after being grafted by PDA/SiO_2_ coating. Hence, the surface energy of CF-PDA/SiO_2_ increases to 62.36 mN·m^−1^, increasing by 14.95% compared to CF-PDA. The improved polar component may be interpreted from the residual amino groups and the introduced hydroxyl groups, and the enhanced dispersion component can be related to the different chemical composition and the increased fiber surface roughness. Hence, the elevated surface energy leads to good interfacial wettability, which can facilitate resin impregnation and the following interfacial reactivity [27].

### 3.3. Composites Interfacial Strength and Hydrothermal Aging Resistance

**Figure 3:**
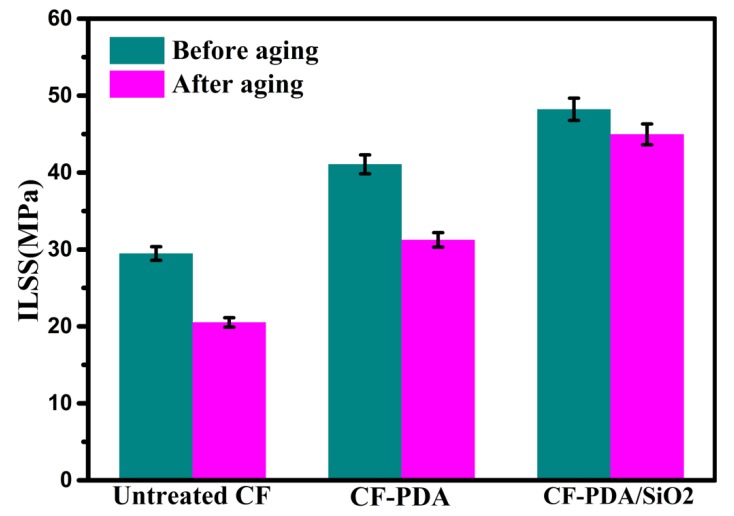
ILSS of composites before and after hydrothermal aging treatment.

ILSS and hydrothermal aging resistance testing results of untreated and modified composites are shown in Figure 3. By comparison with Untreated CF composites, the ILSS value of CF-PDA composites is raised by 39.33% from 29.47 ± 0.88 to 41.06 ± 1.23 MPa because of the effectively activated fiber surfaces and the enhanced resin infiltration after PDA coating. As for CF-PDA/SiO_2_, composites ILSS value is 48.21 ± 1.45 MPa, with the amplification of 63.59% to that of Untreated CF composites. This may be due to that the nanohybrid coating can increase fiber surface energy and the polarity and exhibit a synergistic effect, and thus make the surface easier to be wetted by the hot resin, which are favorable to enhance interfacial interaction and augment mechanical engagement at the interface.

The effectiveness of surface functionalization is also characterized by hydrothermal aging resistance testing. ILSS values of Untreated CF composites have the ILSS retention ratios of 69.63%, indicating a poor hydrothermal aging resistance. By comparison, the increases of the ILSS retention ratios are up to 76.08% for CF-PDA composites and 93.26% for CF-PDA/SiO_2_ composites. The formed nanohybrid coating at the interface region serving as the buffer layer can reduce the number of microcracks for moisture permeation and protect the interface effectively. The significant enhancements of interfacial properties and hydrothermal aging resistance for CF-PDA/SiO_2_ composites confirm that PDA-based modification can produce satisfactory effect in composites properties, which also confirm the successful deposition of PDA and SiO_2_ onto the fiber surface.

### 3.4. Single Fiber Tensile Strength

Single fiber tensile strength (TS) represents its inherent mechanical property and further affects composites in-plane properties. The fiber tensile testing results are presented in Table 2. TS values of CF-PDA and CF-PDA/SiO_2_ are 3.413 ± 0.102 and 3.519 ± 0.105 GPa, respectively, as compared to that of pristine CFs (3.35 GPa). PDA/SiO_2_ nanohybrid coating introduces no deterioration to fiber intrinsic strength due to the mild incubation conditions without bringing fiber surface defects. The novel strategy is to change the fiber surfaces from inert and smooth to polar and rough with avoiding the deterioration of in-plane properties of composites, which can be considered as a powerful alternative approach to carry forward the advanced polymer composites.

## 4. Conclusions

In summary, a uniform mussel-inspired PDA/SiO_2_ layer was successfully deposited onto the fiber surface by a simple and economy strategy of simultaneous polymerization of DA and hydrolysis of TEOS. The attachment of PDA/SiO_2_ nanohybrid coating onto the fiber surface leaded to the improved surface roughness for creating sufficient mechanical interlocking and massive surface polar groups for the increased interfacial wettability compared with those of untreated composites. As a consequence, CF-PDA/SiO_2_ composite simultaneously achieved great advancement in interfacial strength and hydrothermal aging resistance without decreasing fiber intrinsic strength. The ILSS values of composites was improved by 63.59%, and ILSS value of CF-PDA/SiO_2_ reinforced MPSR composites after aging shows a slightly decrease of 6.74%. The promising and green strategy developed in the paper to architecturally build nanohybrid coatings modified fibers with high compatibility and wettability can promote the rapid development and application of advanced polymer composites.

## Figures and Tables

**Table 1:** Surface element analysis of carbon fibers (CFs).

Samples		Element Content (%)				
	C	N	O	Si	O/C	Si/C
Untreated CF	81.61	0.92	17.47	–	0.21	–
CF-PDA	76.21	3.46	20.33	–	0.27	–
CF-PDA/SiO_2_	66.18	2.87	24.12	6.83	0.36	0.10

**Table 2:** Contact angles, surface energy, and tensile strength of different CFs.

Samples	Contact Angles (°)		Surface Energy (mN m^−1^)			TS (GPa)
	*θ*_water_	*θ*_diiodomethane_	*γ* ^d^	*γ* ^p^	*γ*	
Untreated CF	78.50 ± 2.36	58.91 ± 1.77	29.20	6.66	35.86	3.354 ± 0.101
CF-PDA	51.17 ± 1.54	49.89 ± 1.50	34.34	19.91	54.25	3.413 ± 0.102
CF-PDA/SiO_2_	41.89 ± 1.26	40.26 ± 1.21	39.48	22.88	62.36	3.519 ± 0.105
